# Development of a scalable and extendable multi-dimensional health index to measure the health of individuals

**DOI:** 10.1371/journal.pone.0240302

**Published:** 2020-10-07

**Authors:** Chun Wei Yap, Lixia Ge, Reuben Ong, Ruijie Li, Bee Hoon Heng

**Affiliations:** Health Services & Outcomes Research, National Healthcare Group, Singapore, Singapore; University of Auckland, NEW ZEALAND

## Abstract

**Background:**

For population health management, it is important to have health indices that can monitor prevailing health trends in the population. Traditional health indices are generally measurable at different geographical levels with varied number of health dimensions. The aim of this work was to develop and validate a scalable and extendable multi-dimensional health index based on individual data.

**Methods:**

We defined health to be made up of five different domains: Physical, Mental, Social, Risk, and Healthcare utilization. Item response theory was used to develop models to compute domain scores and a health index. These were normalized to represent an individual’s health percentile relative to the population (0 = worst health, 100 = best health). Data for the models came from a longitudinal health survey on 1,942 participants. The health index was validated using age, frailty, post-survey one-year healthcare utilization and one-year mortality.

**Results:**

The Spearman rho between the health index and age, frailty and post-survey one-year healthcare utilization were -0.571, -0.561 and -0.435, respectively, with all p<0.001. The area under the Receiver Operating Characteristic curve (AUROC) for post-survey one-year mortality was 0.930. An advantage of the health index is that it can be calculated using different sets of questions and the number of questions can be easily expanded.

**Conclusion:**

The health index can be used at the individual, program, local, regional or national level to track the state of health of the population. When used together with the domain scores, it can identify regions with poor health and deficiencies within each of the five health domains.

## Background

Health is a multi-dimensional construct and World Health Organization (WHO) had defined health as “a state of complete physical, mental and social well-being and not merely the absence of disease or infirmity” [1]. In order to maintain and improve the health of a population, healthcare systems are increasingly moving upstream and downstream of a hospital-centric system to create a whole system that addresses prevention, early detection, illness care and coordination across settings. In light of these developments, it is important to have health indices that are able to track the changes and monitor prevailing trends in health in the population.

Health indices can measure health at different levels, spanning from individuals to programs, and at local, regional, national, and global levels. At the individual level, individuals could monitor their health through trending of a health index. This could empower them to take charge of their own health better as they could see whether changes in their lifestyle had any impact on their health. At the program level, the effectiveness of the programs could be measured by tracking the change of a health index before and after the implementation of the respective programs. At the regional level, health indices of a population could help in identification of areas with relatively poor health, and specific health interventions could be initiated for different areas according to their health needs. At the national level, longitudinal trending of a health index enables a projection of future health states which can be used to formulate polices to address the projected health needs of the population.

Various approaches for measuring health of population have been documented in literature. Majority of the measures are either summary measures that combine information on mortality and non-fatal health outcomes to represent the health of a particular population as a single number, or measures that describe subjective health states in terms of performance in various domains of health [2]. These measures were developed with conceptualisations ranging from utility, well-being and overall quality of life, through to more narrow conceptualisations like health-related quality of life or health states. Sullivan estimated the disability-free life expectancy by combining mortality and morbidity into a single index [3]. Fanshel developed quality-adjusted life years, which can be used to measure the effects of health programs [4]. Katz calculated the active life expectancy to provide health information at a given population level [5]. This measure can be used to reduce morbidity in the last few years of life by identifying populations for preventive health and medical care. Health-related quality of life includes measures such as EuroQol or SF-36 and covers domains related to physical, mental, emotional and social functioning. It measures the quality-of-life consequences of health status [6]. The Urban Health Index, developed by WHO, measures disparities in health determinants and outcomes in urban area [7]. It can be used to monitor the status of urban areas and to determine the effects of program interventions. The European Deprivation Index evaluates the relative deprivation at a small area level [8]. It is calculated from census aggregated variables that are strongly correlated with individual deprivation indicator and is country-specific. A Child Health Index was created in Sweden from 13 indicators to help monitor children’s health at the local level [9]. Fernandex-Crehuet developed an European Health Index from six dimensions to measure the health status of countries in the European Union [10]. The six dimensions were derived from 29 variables that includes working conditions, general state of health, health system, quality of life, mental health and drug abuse and risk health factors. Zhau constructed a 15-item health index from domains of health conditions, health behaviors and social determinants to measure health changes at county-levels [11]. Santana and colleagues developed a multi-dimensional measure for evaluating health based on equity in health determinants and health outcomes [12]. The Health Determinants and Health Outcomes indices are divided into 10 sub-indices that are areas of concern for population health. The measure was used in all European Union regions and was used to show which regions required investments to reduce health inequalities.

While these summary health indices provide a broad overview of disease-specific health of a particular population, some represent very limited conceptions of health which emphasize a restricted set of physical domains. Some rely on indicators which are available only at a regional level and thus are not informative for individuals’ health states. Other indices such as activities of daily living can be measured at the individual, program, or regional level but these typically only measure a single dimension, or at most two or three dimensions of health. Most of these indices are also not extendable as they can only be calculated from a fixed set of questions or indicators. These questions or indicators could not be substituted with other questions or indicators even if they are mostly similar in the health characteristics that they are measuring. This limits their use, especially over a longitudinal period in different healthcare settings where the use of a consistent set of questions for health measurement could not be guaranteed.

A health index should serve the purpose of:

1. Providing information on the major domains that considered important for health.
2. Comparing across individuals in populations, across populations, and across time.
3. Analysing the benefits of health interventions for use in cost-effectiveness analyses.

The aim of this work is to develop an health index constructed from various objective or subjective instruments with sufficient coverage of important health dimensions and determinants which could provide an overview of the state of health of an individual. This could then be aggregated through averaging or weighted averaging to monitor the health status of a population at a local, regional or national level.

## Methods

We consolidated the ideas behind the International Classification of Functioning, Disability and Health (ICF) framework and other health indices on the methods to describe and measure health and its determinants. In the end, we decided to perceive the health of a person to be made up of five different domains, (1) Physical, (2) Mental, (3) Social, (4) Health risk factors, (5) Healthcare utilization. In this work, we conducted a three-year longitudinal health survey to measure these five health domains.

### Study subjects and sampling method

The survey was conducted from November 2015 to January 2019 on community-dwelling adult population living in the Central region of Singapore. In order to ensure representativeness of the surveyed population, a sampling frame of 5,350 out of 326,500 residential dwelling units in the Central region of Singapore was selected from the National Database on Dwellings in Singapore maintained by the Department of Statistics. The selection technique was stratified design with proportional allocation according to defined broad dwelling type groups (Housing and Development Board properties, condominiums and other apartments, and landed properties). The sample size was calculated using structural equation modelling and based on the rule of thumb of 10 subjects for each item in the instrument, and also accounted for an assumed eligible unit rate of 90%, accessible rate of 80%, response rate of 50%, and 10% dropout rate at year 2 and 3 of the survey. Invitation letters were first sent by post to the selected dwelling units. Trained surveyors then approached the selected dwelling units for recruitment and conducting face-to-face survey. Kish tables were used to identify one household member from each selected dwelling unit to participate in the study [13]. Only Singapore citizens and permanent residents aged 21 years and above, having stayed in the selected household for more than 6 months in the past year were eligible for the survey. Units were treated as not-contactable if the surveyors were unable to get in touch with the selected household member after making three or more visits. A total of 1,942 individuals participated in the survey. The response rate was 53.3%, based on a sample of 3,645 eligible residents in the selected dwelling units. A sample weight was calculated for each participant to adjust for non-response rate, household weight and size, and age-sex distribution in the population. This ensures that analyses based on these survey participants were representative of the estimated 821,650 residents living in the Central region of Singapore. These weights were used in all subsequent modelling and validations. For the purpose of this study, only the first year survey results were used to develop the health index.

This work was approved by the ethical committee of the National Healthcare Group Domain Specific Review Board (Reference Number: 2015/00269). Written informed consent was obtained from each participant after being informed about the study purpose and confidentiality of the data collected.

### Measures

The survey, shown in S1 File, includes eight validated instruments (Modified Barthel Index (MBI) [14], Instrumental Activities of Daily Living (IADL) [15], Late Life Function and Disability Instrument (LLFDI) [16], Mini Nutritional Assessment (MNA) [17], Patient Health Questionnaire (PHQ) [18], Montreal Cognitive Assessment (MoCA) [19], Lubben Social Network Scale [20] and UCLA Loneliness Scale) [21], and 52 questions for socio-demographics (age, gender, ethnicity, nationality, education, employment status, occupation, income level, and housing type), lifestyle, medical conditions, function, symptoms and healthcare utilization.

Physical domain encompasses 13 patient-reported chronic conditions; 4 medical history questions related to back and neck pain, injury and falls; 3 questions for vision, hearing and swallowing difficulties; MBI; IADL; LLFDI; 10 questions on symptoms such as dizziness, nausea; and 3 questions from MNA. Mental domain comprises of 4 patient-reported mental conditions; PHQ; MoCA; UCLA Loneliness Scale; and 2 questions from MNA. Social domain looks at the socioeconomic aspect of a person such as money insufficiency, Lubben Social Network Scale; UCLA Loneliness Scale and 8 questions from LLFDI. Health risk factors domain considers health-related risk behaviors, which may affect a person’s health in the future and were measured using MNA; 5 questions on smoking; and 3 questions on alcohol consumption. Healthcare utilization domain includes number of prescribed medications and historical healthcare utilization of primary care, specialist outpatient clinics, emergency department, inpatient stays, and traditional Chinese medicine practitioners. This last domain is important to help differentiate between treated and untreated health when two persons have similar health based on the earlier four domains.

### Development of health indices

Item response theory (IRT) methodology was used to construct a quantitative measure of health in this work. IRT has been extensively described elsewhere [22, 23] and thus only a brief description is provided here. IRT can be used to measure the latent ability of a person. It is frequently used in education to measure abilities such as mathematical ability that cannot be measured directly but can be estimated indirectly through various questions. IRT determines two parameters for each question, difficulty and discrimination. The difficulty level of a question determines whether a person with a specific level of ability is able to answer the question correctly. A person with higher ability will be able to answer more difficult questions compared to a person with lower ability. The discrimination level of a question determines the probability that a person with lower ability can answer the question correctly. The higher the discrimination level, the lower the probability.

In this work, the latent ability to be measured is a health index of a person. A person with higher health index (i.e. better health) will be able to answer a more ‘difficult’ health question ‘correctly’. For example, a person with higher health index is more likely to answer affirmatively to the question “Are you able to run 800m” compared to a person with poorer health. Also, this question is more ‘difficult’ compared to the question “Are you able to walk around the block”. IRT was used to quantify the difficulty and discrimination for each question used in the longitudinal household survey.

To compute the health index, IRT, implemented in the R package mirt version 1.30, was used to construct a model for each of the five health domains. All the questions in our health survey were first associated to one or more of the five health domains (S1 Table). Questions associated with a health domain were then used to develop an IRT model for that health domain. The IRT model was then used to calculate a theta score for an individual. This theta score represents the health of an individual for that health domain. All the five health domains’ theta score were then averaged to compute a health index for an individual.

**Fig 1:**
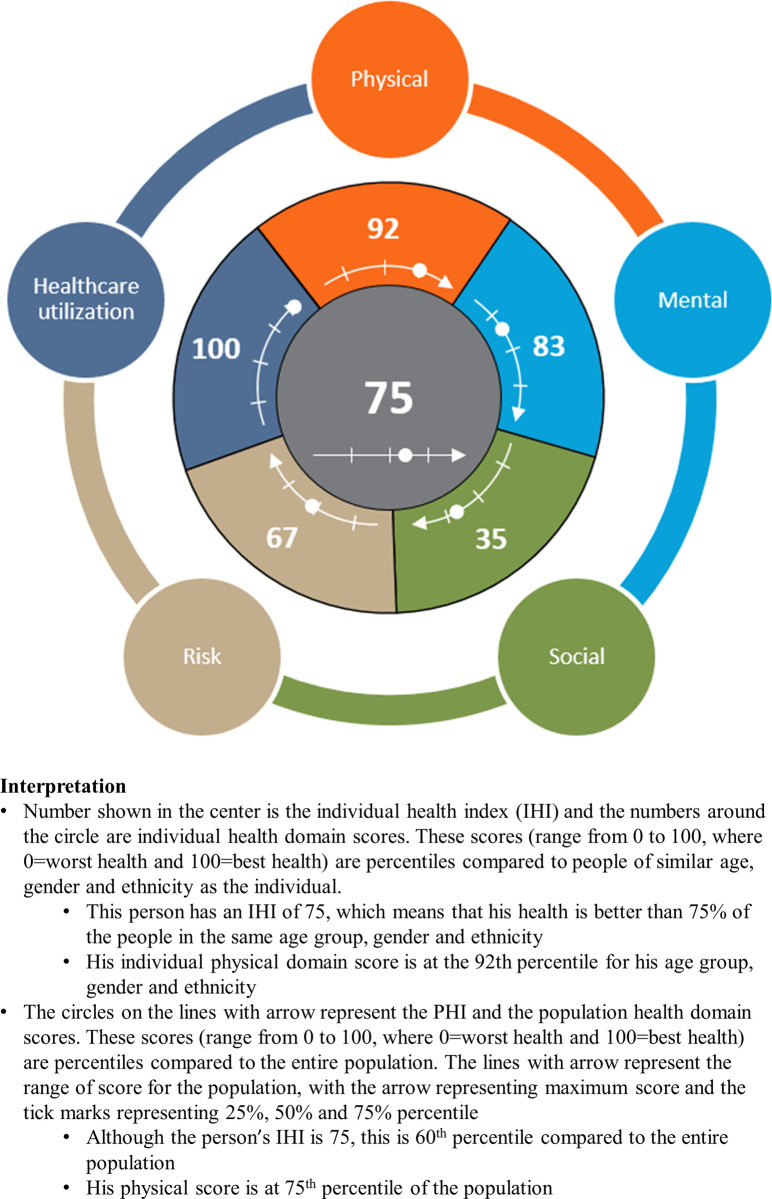
Badge for the IHI, PHI and domain scores for an individual.

To ease interpretation of the health domains’ theta scores and the average theta score, they were normalized to the range 0 to 100 such that 0 represents worst health and 100 represents best health. Two approaches were adopted in this study for the normalization. In the first approach, the distributions of each health domain theta scores and distribution of average theta score for all the survey participants were determined. The percentile of an individual’s domains’ theta scores and average theta score for these distributions were determined and these are known as the population domain scores and population health index (PHI). The population domain scores and PHI will be useful, when aggregated at a regional or national level, for showing the average health of a population. The second approach for normalization determined the distributions of each health domain theta scores and distribution of average theta score for the survey participants for each of the 112 possible strata for age group (21–24, 25–29, 30–34, 35–39, 40–44, 45–49, 50–54, 55–59, 60–64, 65–69, 70–74, 75–79, 80–84, ≥85), gender (male, female), and ethnicity (Chinese, Malay, Indian, Others). The percentile of an individual’s domains’ theta scores and average theta score for the corresponding stratum distributions of that individual were determined and these are known as the individual domain scores and individual health index (IHI). This shows the health at an individual level and allows comparison of the health of an individual relative to similar people in the population. A badge representing the individual and population domain scores, IHI and PHI, and their interpretation is shown in Fig 1.

The validation of the PHI was conducted by correlating with age, frailty, and post-survey one-year healthcare utilization using Spearman correlation, and by predicting one-year mortality. Frailty was determined using the Clinical Frailty Scale (CFS) developed by Rockwood et al [24]. A trained nurse assigned the CFS category for each participant based on their presence of chronic conditions, severity of dementia, performance in activities of daily living, and high-order IADLs (shopping, housekeeping, transportation, handing medication, and finances), frequency of taking part in active recreation or regular fitness program (LLFDI), and presence of active bothersome symptoms [25]. Post-survey one-year healthcare utilization and one-year mortality data were obtained from administrative databases at the Singapore Ministry of Health. Healthcare utilization includes all visits to primary care, specialist outpatient clinics, emergency departments, day surgery and admissions in public healthcare institutions in Singapore. Validation with post-survey one-year healthcare utilization and one-year mortality was restricted to 1,704 participants who had provided additional consent to link their survey data to their healthcare utilization data.

## Results

### Population Health Index (PHI)

In this work, the age of the participants ranged from 21 to 97 years old, with a weighted average age of 49 years. Table 1 shows that the mean PHI decreases as age increases. The Spearman rho between PHI and age is -0.571 (p<0.001). Generally, every 10 years increase in age reduces PHI by 10. The physical, mental, social, risk and utilization health domains generally are reduced by 9, 8, 6, 3 and 7, respectively, for every 10 years increase in age.

**Table 1:** Validation of PHI with age, clinical frailty scale, healthcare utilization and mortality.

	% of participants	Mean PHI (SD)	Mean domain score (SD)				
			Physical	Mental	Social	Risk	Utilization
**Age**							
21 to 34	24.4	68.3 (22.3)	65.8 (23.9)	65.5 (24.5)	62.1 (24.3)	53.3 (27.6)	67.3 (29.9)
35 to 44	18.1	62.0 (26.0)	62.4 (24.7)	60.6 (24.8)	57.6 (26.8)	56.7 (29.1)	60.6 (33.0)
45 to 54	19.1	49.7 (25.2)	53.6 (25.0)	50.9 (26.4)	47.9 (28.1)	48.4 (27.9)	55.1 (33.0)
55 to 64	19.1	41.3 (25.7)	41.6 (25.6)	43.4 (26.9)	44.0 (28.2)	46.3 (29.2)	49.4 (33.9)
65 to 74	11.5	31.4 (23.3)	30.3 (23.7)	33.2 (25.0)	39.6 (28.4)	48.2 (29.8)	38.7 (29.3)
75 to 84	5.9	17.0 (17.1)	15.0 (15.0)	18.0 (19.5)	32.1 (29.3)	42.2 (29.0)	29.9 (23.7)
≥85	1.9	6.7 (7.7)	5.5 (6.4)	9.9 (15.5)	20.7 (25.7)	34.7 (27.4)	30.4 (22.4)
**CFS**							
1. Very fit	15.4	76.3 (19.1)	80.2 (16.0)	66.1 (24.6)	65.1 (25.0)	60.3 (27.4)	78.8 (27.6)
2. Well	36.0	57.5 (24.6)	55.9 (24.6)	55.4 (26.8)	53.7 (27.0)	54.4 (29.1)	65.2 (32.7)
3. Managing well	40.7	40.9 (25.4)	41.4 (24.4)	45.4 (27.7)	46.5 (27.6)	44.7 (27.5)	39.8 (26.6)
4. Vulnerable	3.7	15.8 (16.8)	11.9 (12.1)	24.1 (25.1)	22.9 (25.2)	42.3 (28.5)	35.9 (28.2)
5. Mildly frail	2.0	13.3 (16.1)	10.4 (18.8)	13.4 (14.0)	29.0 (30.3)	42.4 (27.7)	26.5 (21.8)
6. Moderately frail	1.3	4.0 (3.7)	1.9 (0.9)	9.3 (11.3)	16.4 (25.9)	35.3 (29.3)	18.6 (13.1)
7. Severely frail	0.5	2.6 (3.6)	4.5 (6.1)	4.3 (3.1)	13.8 (26.2)	24.2 (22.4)	13.8 (3.8)
8. Very severely frail	0.5	1.5 (1.2)	0.3 (0.5)	31.3 (24.7)	3.4 (2.8)	6.9 (4.4)	34.1 (32.4)
**1-year healthcare utilization**							
S$0	43.0	61.7 (25.5)	50.3 (26.1)	57.9 (27.0)	57.4 (27.4)	55.1 (28.2)	67.7 (32.1)
>S$0 to S$200	8.3	58.1 (25.0)	59.1 (24.7)	54.9 (28.3)	52.6 (28.2)	51.0 (27.4)	64.6 (29.3)
>S$200 to S$500	13.9	50.2 (26.3)	52.3 (27.4)	49.9 (27.7)	51.3 (26.4)	50.4 (28.4)	50.1 (31.5)
>S$500 to S$1,000	12.6	38.9 (26.4)	40.2 (26.1)	42.8 (28.3)	42.6 (28.5)	46.6 (28.3)	39.3 (25.7)
>S$1,000 to S$3,000	14.5	35.4 (27.3)	35.2 (26.2)	41.6 (29.4)	42.6 (29.4)	42.7 (29.3)	36.2 (28.4)
>S$3,000 to S$10,000	3.7	26.8 (26.3)	30.2 (29.5)	35.6 (30.1)	33.1 (31.8)	41.7 (28.7)	28.5 (22.0)
>$10,000	4.1	28.6 (28.6)	24.5 (24.4)	33.1 (27.0)	38.3 (29.7)	39.9 (32.2)	36.8 (31.3)
**1-year mortality**							
Alive	99.1	50.5 (28.8)	50.4 (28.7)	50.4 (29.0)	50.5 (28.9)	50.1 (29.0)	54.2 (33.3)
Dead	0.9	7.4 (5.2)	5.2 (7.1)	16.6 (17.0)	40.5 (38.4)	28.2 (28.8)	28.8 (27.6)

Most of the participants (92.1%) had Clinical Frailty Scale (CFS) 1 to 3, with 5.7% having CFS 4 to 5 and 2.3% having CFS 6 to 8. The mean PHI decreases as the CFS increases (Table 1) with a Spearman rho of -0.561 (p<0.001). This moderate correlation between PHI and CFS is expected since CFS mainly considers only the physical and mental domains whereas PHI considers these two plus three other health domains.

Post-survey one-year healthcare utilization among those who are alive ranged from zero to S$131,008, with a median spending of S$216. A total of 663 participants had zero spending and 1,025 participants had non-zero spending. Table 1 shows that the Spearman rho between PHI and 1-year healthcare utilization is -0.435 (p<0.001). A univariate logistic regression shows that the likelihood of a non-zero spending increases by 29% (p<0.001) for every 10 unit decrease in PHI. Among those with non-zero spending, a univariate gamma regression with log link function shows that the spending increases by 12% (p = 0.001) for every 10 unit decrease in PHI.

A total of 16 (0.9%) participants died within 1 year of the survey. The median age at death is 75 (range: 53 to 93) and the median PHI is 6 (range: 1 to 19). Using PHI to predict 1-year mortality had an area under the Receiver Operating Characteristic curve (AUROC) of 0.930 (CI: 0.929 to 0.930).

**Fig 2:**
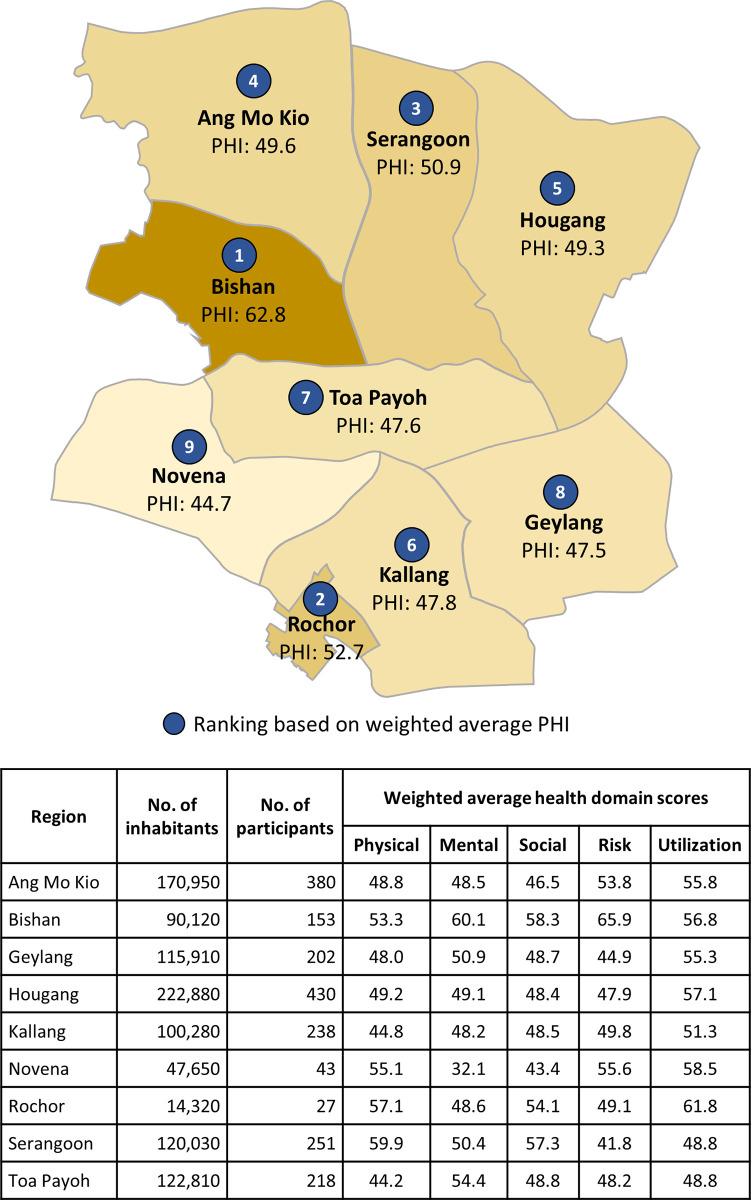
Weighted average PHI for each of the nine planning areas in the Central region of Singapore.

Fig 2 shows the weighted average PHI for each of the nine planning areas in the Central region of Singapore. The results suggest that people living in Bishan generally had better health while people living in Novena generally had poorer health compared to people living elsewhere in the Central region of Singapore. Analysis of the population health domain scores show that people living in Novena had poorer health in both Mental and Social domains. Hence, population health efforts to improve health in Novena should direct more of their efforts in these health domains. For people living in Serangoon, although their health is generally better than most of the other areas, they had the poorest score in Risk domain. Population health efforts should concentrate on reducing the risky lifestyle behaviors (smoking, drinking and poor nutrition) of residents living in Serangoon to further improve their health.

### Individual Health Index (IHI)

To demonstrate the use of IHI, we will also present two case studies. Participant 3535 is a 61 years old female with an IHI of 79 during the first interview. This increased to 91 at the second interview in the second year and further increased to 100 at the third interview in the third year. Analyses of her individual domain scores show that she had a poor Physical domain score of 55 initially. At the third year, she had improved her Physical domain score to 100, mainly due to improvement in the scores for the Late Life Function and Disability Instrument. Participant 5002 is a 36 years old male with an IHI of 81 during the first interview. This decreased to 75 at the second interview in the second year and further decreased to 68 at the third interview in the third year. Analyses of his individual domain scores show that he had a good Mental domain score of 85 initially. At the third year, his Mental domain score decreased to 29, mainly due to poorer scores in his Montreal Cognitive Assessment short term memory and executive areas.

## Discussion

In this work, we developed two health indices (PHI and IHI) to determine the state of health of an individual. PHI compares a person’s health with that of an entire population. Since it is expected that young people will generally have better health than older people, PHI is not useful at an individual level. Rather, by calculating the average or weighted average PHI of a representative group of individuals, it can be used to monitor the state of health of a population at a regional or national level. IHI compares a person’s health with people of similar age, gender and ethnicity. Hence, it is more useful at an individual level for people to monitor their health at various time points.

The two health indices take into account five different health domains. Some may ask, “why five domains?” After all, when we say a person is in good health, we usually mean that the person is healthy both physically and mentally. Similarly, when a person is in poor health, typically we think of physical weakness or with multiple chronic illnesses. Social factors, health risk factors and healthcare utilization are seldom considered as part of a person’s health. Yet, it is important to consider these factors. Dalgreen and Whitehead had shown that social determinants affect health [26]. Health risk factors such as smoking, drinking, obesity were widely recognized to result in ill health [27]. Healthcare utilization is intended to differentiate between natural versus treated health. For example, two persons may be able to perform the same physical activities, but one can do that without any medical treatment whereas the other requires medical treatment. Thus, if we just rely on measuring the physical domain, we might think the two had the same level of health. However, by including a healthcare utilization domain, we would be able to assign a lower level of health to the person with medical treatments.

Our results show that there is an inverse relationship between PHI and age. This is in concordance with the typical observations that the older age group tend to have more physical or mental conditions and thus poorer health compared to the younger age group. Our results also show that there is an inverse relationship between PHI and healthcare utilization. It is important to note that while a health index should correlate with healthcare utilization, the correlation may not necessarily be high because healthcare utilization is dependent not only on the health of a person. It is also dependent on other factors such as the healthcare seeking behavior of a person. A person with good physical health might choose to go for yearly check-ups and tests while a person with poor physical health might decide to forgo treatment due to disbelief in the treatment or inability to afford the costs. Healthcare utilization is also affected by unpredictable factors that are unrelated to the health of a person, such as accidents.

The health indices developed in this work have several advantages. Firstly, PHI can be used at the regional or national level to track the state of health of the population. When used together with the population domain scores, it can help identify regions with poor health and the deficiencies within each of the five health domains. This facilitates the delivery of appropriate interventions. PHI can also be used as an outcome measure to evaluate the effectiveness of healthcare intervention programs in improving the health of the population. IHI can be used to help individuals manage their own health. When measured across multiple time-points, individuals will be able to know whether their health is improving or deteriorating, and whether their lifestyle changes or medical treatments are having any effects on their health.

The advantage of using IRT to develop models for calculating the health indices is that once the difficulty and discrimination has been determined for each question, questions with similar difficulty and discrimination can be substituted for one another. This means that we are no longer restricted to a standard set of questions for measuring the health of a person. We could mix and match different sets of questions together as long as each set consists of questions with similar difficulty and discrimination ranges. This provides implementation flexibility as different sets of questions can be chosen to suit different needs and workflow of the individual healthcare groups. In the event that the existing set of questions are not suitable or sufficient for the needs and workflow, new questions can be added to the existing pool of questions through a calibration process that involves administering the new questions with some existing questions to a group of individuals [22, 23]. This ability to add new questions enables the health indices to maintain their comparability across different people and time when new instruments are developed in the future. It also allows the health indices to be calculated from retrospective survey data once those questions have been calibrated.

Currently, we have a question bank of 265 questions (S1 Table). From these, we have identified a set of 64 questions that can be used to calculate the health indices for those using traditional paper-and-pencil survey (S1 Table). We have also developed a computerized adaptive testing system using the R package mirtCAT version 1.8. This system chooses the questions to be asked based on the person’s responses to earlier questions so that the number of questions used will be minimized and the type of questions will be customized to the person’s health. This ability to adapt to previous responses makes it suitable for use in electronic medication records (EMR) where the system will extract relevant information from the EMR and only choose additional questions if the EMR does not have sufficient information to compute the health indices.

This work has several limitations. The health of a person is preconceived to be made up of five health domains. It can be argued that these domains appear to be arbitrarily chosen and lack validity other than face validity. In some indices, such as NEO-PI [28], the dimensions were empirically derived directly from the data. Future studies could explore factor analysis or hierarchical clustering of the questions in this work to determine the dimensions. Another limitation is the level of analysis problem. It is not clear whether the same categorizations and measures can be applied to the health of individual persons, and of groups/organizations/nations, without committing 'ecological fallacies' in the statistical reasoning. What is true for individuals is at a different level of analysis and has different behavioral dynamics than what is true for social groups, organizations, and societies. In this work, PHI, IHI and their respective domain scores were normalized to a defined population, which may not be representative of other populations. Thus, different countries will need to define their own population for normalization of the health indices and domain scores. Another limitation is that PHI and IHI were calculated from a simple average of the domain scores. A weighted average may be better since some questions might contribute to more than one health domain. However, it is difficult to determine appropriate weights for each health domain since there is no gold standard for health.

## Conclusion

Two scalable and extendable multi-dimensional health indices were developed in this work. These health indices are composite measures of five different health domains, namely physical, mental, social, health risk factors and healthcare utilization. They are scalable as they are suitable for use at the individual, program, regional and national level to measure the state of health. As the health indices were developed using IRT, their calculations are not limited to a standard set of questions. Instead, different sets of questions can be used and the number of questions can be easily expanded with a calibration process. This extendable property will enable the calculation of the health indices over a longitudinal period in different healthcare settings where the use of a consistent set of questions for health measurement could not be guaranteed. This is a major advantage over existing population health measures.

Other than the two health indices, the health domain scores that are associated with each health index can be used to identify specific domains of good and poor health. This facilitates targeted interventions into the specific health domains of concern.

## Supporting information

S1 File Survey questionnaire. (DOCX)

S1 Table Question bank and their mapping to the five health domains. (XLSX)
