# Supplementary material for: Development of a scalable and extendable multi-dimensional health index to measure the health of individuals
Source: PLoS One. 2020 Oct 7;15(10):e0240302. doi: 10.1371/journal.pone.0240302 (PMC7540893; doi:10.1371/journal.pone.0240302)
Supplement: S1 File — (DOCX) [file pone.0240302.s001.docx]

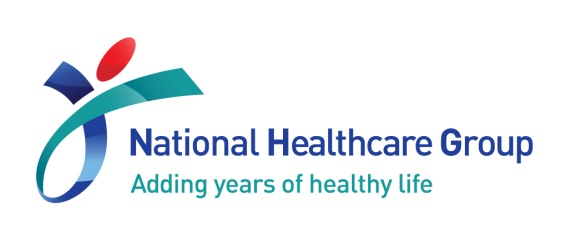


Reference No. ⬜ ⬜ ⬜ ⬜ - ⬜ ⬜

Population Health Survey

| **Sections** | **Page(s)** | **Sections** | **Page(s)** |
| --- | --- | --- | --- |
|  |  |  |  |
| 1. **DEMOGRAPHICS** | 1 | 1. **HEALTHCARE UTILISATION & MEDICATION** | 21 |
|  |  |  |  |
| 1. **MEDICAL HISTORY** | 4 | 1. **MENTAL HEALTH** 2. Patient Health Questionnaire (Depression & Anxiety) | 22 |
|  |  |  |  |
| 1. **LIFESTYLE & ENVIRONMENT** | 7 | 1. **COGNITION** 2. Montreal Cognitive Assessment | 24 |
|  |  |  |  |
| 1. **FUNCTIONAL STATUS** | 8 | 1. **SOCIO-ECONOMIC STATUS** | 25 |
|  |  |  |  |
| 1. **ACTIVITIES OF DAILY LIVING** 2. Modified Barthel Index 3. Instrumental Activities of Daily Living 4. Late Life Function and Disability Instrument | 9  13  15 | 1. **SOCIAL** 2. Social Isolation 3. Loneliness | 25  25 |
|  |  |  |  |
| 1. **SYMPTOMS** | 19 | 1. **QUALITY OF LIFE** 2. EQ-5D-5L | 26 |
|  |  |  |  |
| 1. **NUTRITION** 2. Mini Nutritional Assessment | 20 |  |  |

| Date of interview : |  |
| --- | --- |

Reference No. ⬜ ⬜ ⬜ ⬜ - ⬜ ⬜ Self-reported □ Proxy* □

*The proxy is to rate how he/she (the proxy) thinks the respondent would rate his/her own responses if he/she (the respondent) was able to communicate it.

# Demographics

|  |  |  | | |  |
| --- | --- | --- | --- | --- | --- |
| **Age** | : |  |  |  | |
|  |  |  |  |  | |
| **Gender** | : | □ | 1) Male |  | |
|  |  | □ | 2) Female |  | |

|  |  | **1) Single** | **2) Married** | **3) Cohabiting** | **4) Widowed** | **5) Divorced /**  **Separated** |
| --- | --- | --- | --- | --- | --- | --- |
| **Marital status** | : | □ | □ | □ | □ | □ |

| **Ethnicity** | : | | | □ | | | | 1) Chinese | | | | | |
| --- | --- | --- | --- | --- | --- | --- | --- | --- | --- | --- | --- | --- | --- |
|  |  | | | □ | | | | 2) Malay | | | | | |
|  |  | | | □ | | | | 3) Indian | | | | | |
|  |  | | | □ | | | | 4) Others: |  | |  | | |
|  | |  | | |  | | | | |  | |  |  |
| **Employment status** | | | : | | |  | 1) Employed: □Full-time □Part-time | | | | | |  |
|  | | |  | | |  | 2) Unemployed: □Voluntary □Involuntary | | | | | |  |
|  | | |  | | |  | 3) Inactive: □Homemaker □ Retired  □Non-paid □Student / National Service | | | | | |  |
|  | | |  | | |  | 4) Permanently sick/unfit for work | | | | | |  |

| **Occupation** | : | Job title: | |  |  |
| --- | --- | --- | --- | --- | --- |
|  |  |  | |  |  |
|  |  | □ | 1) Legislators, Senior Officials and Managers | | |
|  |  | □ | 2) Professionals | | |
|  |  | □ | 3) Associate Professionals and Technicians | | |
|  |  | □ | 4) Clerical Support Workers | | |
|  |  | □ | 5) Service and Sales Workers | | |
|  |  | □ | 6) Agricultural and Fishery Workers | | |
|  |  | □ | 7) Craftsmen and Related Trades Workers | | |
|  |  | □ | 8) Plant and Machine Operators and Assemblers | | |
|  |  | □ | 9) Cleaners, Labourers and Related Workers | | |
|  |  | □ | 10) Workers not Classifiable by Occupation | | |
|  |  | □ | 11) Others | | |

| **Nationality** | : | □ | 1) Singaporean |  |
| --- | --- | --- | --- | --- |
|  |  | □ | 2) Singapore PR |  |
|  |  |  |  |  |
| **Highest Education**  **Attained** | : | □ | 0) No formal qualification/Pre-Primary/Lower Primary  (Primary education without PSLE/PSPE or equivalent; Certificate in BEST 1-3) | |
|  |  | □ | 1) Primary  (Certificate in BEST 4; PSLE/PSPE or equivalent; at least 3 WSQ Statements  of attainment in WPLN at Level 1 or 2) | |
|  |  | □ | 2) Lower Secondary  (Secondary education without a GCE ‘O’/’N’ Level pass or their equivalent;  Certificate in WISE 1-3; Basic vocational certificates; at least 3 WSQ  Statements of attainment in WPLN at Level 3 or 4) | |
|  |  | □ | 3) Secondary  (‘N’ Level; ‘O’ Level; Nitec (intermediate) or equivalent; ISC or equivalent;  at least 3 WSQ Statements of attainment in WPLN at Level 5 and above) | |
|  |  | □ | 4) Post-Secondary  (‘A’/’H2’ Level / Nitec or equivalent/WSQ Certificate or equivalent) | |
|  |  | □ | 5) Polytechnic Diploma | |
|  |  | □ | 6) Professional Qualification and other diploma  (ITE/NIE/SIM/LaSalle-SIA/NAFA/WSQ diploma or equivalent) | |
|  |  | □ | 7) Bachelor’s Degree or equivalent | |
|  |  | □ | 8) Postgraduate Diploma/ Certificate | |
|  |  | □ | 9) Master’s and Doctorate or equivalent | |

| **Personal Income** | : | □ | 1) $1,000 or less |
| --- | --- | --- | --- |
| **(Monthly)** |  | □ | 2) $1,001 - $1,500 |
|  |  | □ | 3) $1,501 - $2,000 |
|  |  | □ | 4) $2,001 - $2,500 |
|  |  | □ | 5) $2,501 - $3,000 |
|  |  | □ | 6) $3,001 - $4,000 |
|  |  | □ | 7) $4,001 -,$5,000 |
|  |  | □ | 8) $5,001 -$6,000 |
|  |  | □ | 9) More than $6,000 |

| **Housing Type** | : | □ | 1) HDB Studio Apartment | | |
| --- | --- | --- | --- | --- | --- |
|  |  | □ | 2) HDB 1-2 room flat | | |
|  |  | □ | 3) HDB 3 room flat | | |
|  |  | □ | 4) HDB 4 room flat | | |
|  |  | □ | 5) HDB 5 room flat/ Executive/ 3-Generation family flat | | |
|  |  | □ | 6) Maisonette / HUDC | | |
|  |  | □ | 7) Private Apartment / Condominium | | |
|  |  | □ | 8) Landed Property | | |
|  |  | □ | 9) Others: |  |  |

| **Living arrangement** | : | □ | 1) Alone |
| --- | --- | --- | --- |
|  |  | □ | 2) With spouse |
|  |  | □ | 3) With children or grandchildren |
|  |  | □ | 4) With other relatives or friends |
|  |  | □ | 5) With other unrelated individuals: ____________________ |

# Medical history

| **1.** | **Have you ever been told to have any of these conditions by a Western-trained doctor?** | **0) No** | **1) Yes** | **2) Do not**  **know** |
| --- | --- | --- | --- | --- |
|  | Diabetes / gestational diabetes | □ | □ | □ |
|  | High blood pressure | □ | □ | □ |
|  | High blood cholesterol | □ | □ | □ |
|  | Overweight / obesity | □ | □ | □ |
|  | Heart attack | □ | □ | □ |
|  | Heart failure | □ | □ | □ |
|  | Stroke / Transient Ischaemic attacks | □ | □ | □ |
|  | Asthma | □ | □ | □ |
|  | Chronic bronchitis / emphysema / COPD | □ | □ | □ |
|  | Chronic kidney disease | □ | □ | □ |
|  | Cancer (Please specify: ) | □ | □ | □ |
|  | Osteoarthritis / gout / rheumatoid arthritis | □ | □ | □ |
|  | Osteoporosis | □ | □ | □ |
|  | Depression | □ | □ | □ |
|  | Anxiety disorder | □ | □ | □ |
|  | Dementia / Alzheimer’s | □ | □ | □ |
|  | Schizophrenia | □ | □ | □ |
|  | Parkinson’s disease | □ | □ | □ |

|  | |  | **GP /**  **Polyclinic** | | **Specialist** | | | | | | | **TCM / Others** | | | | **Not Applicable** |  |
| --- | --- | --- | --- | --- | --- | --- | --- | --- | --- | --- | --- | --- | --- | --- | --- | --- | --- |
|  | | **In the past 6 months, have you ever sought medical consultation / treatment for…?** | **0) No** | **1) Yes** | **0) No** | | | **1) Yes** | | | | **0) No** | | | **1) Yes** |  |  |
|  | | Chronic low back pain (>3 months) | □ | □ | □ | | | □ | | | | □ | | | □ | □ |  |
|  | | Chronic neck pain (>3 months) | □ | □ | □ | | | □ | | | | □ | | | □ | □ |  |
|  |  | | | | | | **0) No** | | | **1) Yes** | | |  |  |  |  |  |
|  | **Have you ever had a major injury resulted in amputation, skeletal injuries, burns, injuries to internal organs, eye injuries, unconsciousness or acute illness due to biochemical agent exposure?** | | | | | | □ | | | □ | | |  |  |  |  |  |
|  |  | | | | | **0) No** | | | **1) Yes** | | **If yes, how many times?** | | | | | |  |
|  | **Did you have a fall in the past 6 months?**  (exclude “near misses” and “assisted falls”) | | | | | □ | | | □ | |  | | | | | |  |
|  | **If ‘No’ was checked for Q4, please go to Q6:** | | | | | **0) No** | | | **1) Yes** | |  | | | | | |  |
|  | **Did the fall have any significant impact on your daily living in the past 6 months?** | | | | | □ | | | □ | |  | | | | | |  |

# Lifestyle and Environment

|  |  | **0) No** | **1) Yes** | **If yes, how long would 1 pack (20 sticks) last you?** | |
| --- | --- | --- | --- | --- | --- |
|  | **Do you currently smoke tobacco products?** | □ | □ |  | days |

**If “Yes” was checked for Q6, please go to Q8:**

|  | |  | **0) No** | **1) Yes** | | **If yes, how many years of smoking before you quitted?** | | | | | | |
| --- | --- | --- | --- | --- | --- | --- | --- | --- | --- | --- | --- | --- |
|  | | **Have you ever smoked tobacco products in the past?** | □ | □ | |  | | | | | years | |
|  |  | | | | **1) Very often** | | **2) Often** | **3) Once in**  **a while** | **4) Almost never** | **5) Never** | |  |
|  | **How often are you exposed to second-hand smoke in indoor spaces such as home, workplace, restaurants, cars, etc.?** | | | | □ | | □ | □ | □ | □ | |  |
|  |  | | | | **0) Never** | | **1) Monthly or less** | **2) 2-4 times a month** | **3)2-3 times a week** | **4) ≥ 4 times a week** | |  |
|  | **How often do you have a drink^1^ containing alcohol?** | | | | □ | | □ | □ | □ | □ | |  |

**If ‘Never’ was checked for Q9, please go to Q12 :**

|  |  | **0) 1 or 2** | **1) 3 or 4** | **2) 5 or 6** | **3) 7 to 9** | **4) 10 or**  **more** |
| --- | --- | --- | --- | --- | --- | --- |
|  | **How many drinks^1^ containing alcohol do you have on a typical day when you are drinking?** | □ | □ | □ | □ | □ |

|  |  | **0) Never** | **1) Less than monthly** | **2) Monthly** | **3) 2-3 times per week** | **4) ≥ 4 times a week** |
| --- | --- | --- | --- | --- | --- | --- |
|  | **How often do you have six or more drinks^1^ on one occasion?** | □ | □ | □ | □ | □ |

*^1^ One alcoholic drink refers to 1 can/small bottle (~285mls) of beer or 1 glass (~120mls) of wine or 1 measure (~30mls) of spirits*

# Functional Status

|  | | **0) No** | **1) Yes** |
| --- | --- | --- | --- |
|  | **Do you have difficulties with vision, even with glasses?** | □ | □ |
|  | **Do you have difficulty with hearing, even if you use a hearing aid?** | □ | □ |
|  | **Do you have any problems in chewing or swallowing your food?** | □ | □ |

# Activities of daily living

## Modified Barthel Index (MBI)

|  | **Personal hygiene**  ① Unable to attend to personal hygiene and is dependent in all aspects.  ② Assistance is required in all steps of personal hygiene.  ③ Some assistance is required in one or more steps of personal hygiene.  ④ Able to conduct his/her own personal hygiene but requires minimal assistance before and/or after the operation.  ⑤ Can wash his/her hands and face, comb hair, clean teeth and shave. A male may use any kind of razor but must insert the blade, or plug in the razor without help, as well as retrieve it from the drawer or cabinet. A female must apply her own make-up, if used, but need not braid or style her hair. |
| --- | --- |
|  | **Bathing self**  ① Total dependence in bathing self.  ② Assistance is required in all aspects of bathing.  ③ Assistance is required with either transfer to shower/bath or with washing or drying; including inability to complete a task because of condition or disease etc.  ④ Supervision is required for safety in adjusting the water temperature, or in the transfer.  ⑤ May use a bath tub, a shower, or take a complete sponge bath. The patient must be able to do all the steps of whichever method is employed without another person being present. |
|  | **Feeding**  ① Dependent in all aspects and needs to be fed.  ② Can manipulate an eating device, usually a spoon, but someone must provide active assistance during the meal.  ③ Able to feed self with supervision. Assistance is required with associated tasks such as putting milk/sugar into tea, salt, pepper, spreading butter, turning a plate or other “set-up” activities.  ④ Independence in feeding with prepared tray except may be cut meat, open milk carton, jar lid etc. Presence of another person is not required.  ⑤ Can feed self from a tray or table when someone puts the food within reach. Must put on an assistive device if needed, cut the food, and if desired, use salt and pepper, spread butter, etc. |
|  | **On and off the toilet**  ① Fully dependent in toileting.  ② Assistance required in all aspects of toileting.  ③ Assistance may be required with management of clothing, transferring, or washing hands.  ④ Supervision may be required for safety with normal toilet. A commode may be used at night but assistance is required for emptying and cleaning.  ⑤ Able to get on and off the toilet, fasten and unfasten clothes, prevent soiling of clothes and use toilet paper without help. If necessary, the person may use a bed pan or commode, or urinal at night, but must be able to empty it, and clean it. |
|  | **Stairs**  ① Unable to climb stairs.  ② Assistance is required in all aspects of stair climbing, including assistance with walking aids.  ③ Able to ascend/descend but is unable to carry walking aids, and needs supervision and assistance.  ④ Generally no assistance is required. At times supervision is required for safety due to morning stiffness, shortness of breath etc.  ⑤ Able to go up and down a flight of stairs safely without help or supervision. Able to use hand rails, cane, or crutches when needed and is able to carry these devices as he/she ascends or descends. |
|  | **Dressing**  ① Dependent in all aspects of dressing and is unable to participate in the activity.  ② Able to participate to some degree, but is dependent in all aspects of dressing.  ③ Assistance is needed in putting on, and/or removing any clothing.  ④ Only minimal assistance is required with fastening clothing, such as buttons, zips, bra, shoes etc.  ⑤ Able to put on, remove, and fasten clothing, tie shoelaces, or put on, fasten, remove corset, braces, as prescribed. |
|  | **Bowels**  ① The person is bowel incontinent.  ② Needs help to assume appropriate position, and with bowel movement facilitatory techniques.  ③ Can assume appropriate position, but cannot use facilitatory techniques, or clean self without assistance and has frequent accidents. Assistance.  ④ May require supervision with the use of suppository or enema and has occasional accidents.  ⑤ Can control bowels and has no accidents, can use suppository, or take an enema when necessary. |
|  | **Bladder**  ① Dependent in bladder management, is incontinent, or has indwelling catheter.  ② Incontinent but is able to assist with the application of an internal or external device.  ③ Generally dry by day, but not at night, and needs some assistance with the devices.  ④ Generally dry by day and night, but may have an occasional accident, or need minimal assistance with internal or external devices.  ⑤ Able to control bladder day and night, and/or is independent with internal or external devices. |
|  | **Chair/bed transfers**  ① Unable to participate in a transfer. Two attendants are required to transfer the person with or without a mechanical device.  ② Able to participate but maximum assistance of one other person is required in all aspects of the transfer.  ③ The transfer requires the assistance of one other person. Assistance may be required in any aspect of the transfer.  ④ The presence of another person is required either as a confidence measure, or to provide supervision for safety.  ⑤ Can safely approach the bed in a wheelchair, lock the brakes, lift the footrests, move safely to bed, he down, come to a sitting position on the side of the bed, change the position of the wheelchair, transfer back into it safely. Must be independent in all phases of this activity. |
|  | **Ambulation**  ① Dependent in ambulation.  ② Constant presence of one or more assistants is required during ambulation.  ③ Assistance is required with reaching aids and/or their manipulation. One person is required to offer assistance.  ④ Independent in ambulation but unable to walk 50 yards/metres without help, or supervision is needed for confidence or safety in hazardous situations.  ⑤ Must be able to wear braces if required, lock and unlock these braces, assume standing position, sit down, and place the necessary aids into position for use. Must be able to use crutches, canes, or a walker, and walk 50 metres/ yards without help or supervision. |
|  | **Wheelchair management (alternative to Ambulation)** |

Only use this item if the person is rated “①” for Ambulation, and then only if he/she has been trained in wheelchair management

① Dependent in wheelchair ambulation.

② Can propel self short distances on flat surface, but assistance is required for all other steps of wheelchair management.

③ Presence of one person is necessary and constant assistance is required to manipulate chair to table, bed etc.

④ Can propel self for a reasonable duration over regularly encountered terrain. Minimal assistance may still be required in “tight comers”.

⑤ To propel wheelchair independently, the person must be able to go around comers, turn around, manoeuvre the chair to a table, bed, toilet, etc. The person must be able to push a chair at least 50 metres/yards.

# Instrumental Activities of Daily Living (IADL)

| 1. **Ability to Use Telephone**   ① Operates telephone on own initiative; looks up and dials numbers.  ② Dials a few well-known numbers.  ③ Answers telephone, but does not dial.  ④ Does not use telephone at all. |
| --- |
| 1. **Shopping**   ① Takes care of all shopping needs independently.  ② Shops independently for small purchases.  ③ Needs to be accompanied on any shopping trip.  ④ Completely unable to shop. |
| 1. **Food Preparation**   ① Plans, prepares, and serves adequate meals independently.  ② Prepares adequate meals if supplied with ingredients.  ③ Heats and serves prepared meals or prepares meals but does not maintain adequate diet.  ④ Needs to have meals prepared and served. |
| 1. **Housekeeping**   ① Maintains house alone with occasion assistance (heavy work).  ② Performs light daily tasks such as dishwashing, bed making.  ③ Performs light daily tasks, but cannot maintain acceptable level of cleanliness.  ④ Needs help with all home maintenance tasks.  ⑤ Does not participate in any housekeeping tasks. |
| 1. **Laundry**   ① Does personal laundry completely.  ② Launders small items, rinses socks, stockings, etc.  ③ All laundry must be done by others. |
| 1. **Mode of Transportation**   ① Travels independently on public transportation or drives own car.  ② Arranges own travel via taxi, but does not otherwise use public transportation.  ③ Travels on public transportation when assisted or accompanied by another.  ④ Travel limited to taxi or automobile with assistance of another.  ⑤ Does not travel at all. |
| 1. **Responsibility for Own Medications**   ① Is responsible for taking medication in correct dosages at correct time.  ②Takes responsibility if medication is prepared in advance in separate dosages.  ③ Is not capable of dispensing own medication. |
| 1. **Ability to Handle Finances**   ① Manages financial matters independently (budgets, writes checks, pays rent and bills, goes to bank); collects and keeps track of income.  ② Manages day-to-day purchases, but needs help with banking, major purchases, etc.  ③ Incapable of handling money. |

# Late Life Function and Disability Instrument (LLFDI)

Disability Questions

|  | |  | **How often do you…?** | | | | | **To what extent do you feel limited in…?** | | | | |
| --- | --- | --- | --- | --- | --- | --- | --- | --- | --- | --- | --- | --- |
|  | |  | **5) Very Often** | **4) Often** | **3) Once in a While** | **2) Almost Never** | **1) Never** | **5) Not at All** | **4) Little** | **3) Somewhat** | **2) A Lot** | **1) Completely** |
|  | Keep (Keeping) in touch with others through letters, phone, or email. | | □ | □ | □ | □ | □ | □ | □ | □ | □ | □ |
|  | Visit (Visiting) friends and family in their homes. | | □ | □ | □ | □ | □ | □ | □ | □ | □ | □ |
|  | Provide (Providing) care or assistance to others. This may include providing personal care, transportation, and running errands for family members or friends. | | □ | □ | □ | □ | □ | □ | □ | □ | □ | □ |
|  | Take (Taking) care of the inside of your home. This includes managing and taking responsibility for homemaking, laundry, housecleaning and minor household repairs. | | □ | □ | □ | □ | □ | □ | □ | □ | □ | □ |
|  | Work (Working) at a volunteer job outside your home. | | □ | □ | □ | □ | □ | □ | □ | □ | □ | □ |
|  | Take (Taking) part in active recreation. This may include bowling, golf, tennis, hiking, jogging, or swimming. | | □ | □ | □ | □ | □ | □ | □ | □ | □ | □ |
|  | Take (Taking) care of household business and finances. This may include managing and taking responsibility for your money, paying bills, dealing with a landlord or tenants, dealing with utility companies or governmental agencies. | | □ | □ | □ | □ | □ | □ | □ | □ | □ | □ |
|  |  | | **How often do you…?** | | | | | **To what extent do you feel limited in…?** | | | | |
|  |  | | **5) Very Often** | **4) Often** | **3) Once in a While** | **2) Almost Never** | **1) Never** | **5) Not at All** | **4) Little** | **3) Somewhat** | **2) A Lot** | **1) Completely** |
|  | Take (Taking) care of your own health. This may include managing daily medications, following a special diet, scheduling doctor’s appointments. | | □ | □ | □ | □ | □ | □ | □ | □ | □ | □ |
|  | Travel (Traveling) out of town for at least an overnight stay. | | □ | □ | □ | □ | □ | □ | □ | □ | □ | □ |
|  | Take (Taking) part in a regular fitness program. This may include walking for exercise, stationary biking, weight lifting, or exercise classes. | | □ | □ | □ | □ | □ | □ | □ | □ | □ | □ |
|  | (Inviting) people into your home for a meal or entertainment. | | □ | □ | □ | □ | □ | □ | □ | □ | □ | □ |
|  | Go (Going) out with others to public places such as restaurants or movies. | | □ | □ | □ | □ | □ | □ | □ | □ | □ | □ |
|  | Take (Taking) care of your own personal care needs. This includes bathing, dressing, and toileting. | | □ | □ | □ | □ | □ | □ | □ | □ | □ | □ |
|  | Take (Taking) part in organized social activities. This may include, clubs, card playing, senior center events, community or religious groups. | | □ | □ | □ | □ | □ | □ | □ | □ | □ | □ |
|  | Take (Taking) care of local errands. This may include managing and taking responsibility for shopping for food and personal items, and going to the bank, library, or dry cleaner. | | □ | □ | □ | □ | □ | □ | □ | □ | □ | □ |
|  | Prepare (Preparing) meals for yourself. This includes planning, cooking, serving, and cleaning up. | | □ | □ | □ | □ | □ | □ | □ | □ | □ | □ |

Function Questions

|  | **How much difficulty do you have…?**  **(Remember, this is without the help of someone else and without the use of any assistive walking device.)** | **5) None** | **4) A Little** | **3) Some** | **2) Quite a Lot** | **1) Cannot do** |
| --- | --- | --- | --- | --- | --- | --- |
|  | Unscrewing the lid off a previously unopened jar without using any devices | □ | □ | □ | □ | □ |
|  | Going up and down a flight of stairs using a handrail | □ | □ | □ | □ | □ |
|  | Putting on and taking off long pants (including managing fasteners) | □ | □ | □ | □ | □ |
|  | Running 800 meters or more | □ | □ | □ | □ | □ |
|  | Using common utensils for preparing meals (e.g., can opener, potato peeler, or sharp knife) | □ | □ | □ | □ | □ |
|  | Holding a full glass of water in one hand | □ | □ | □ | □ | □ |
|  | Walking 1.6 kilometres, taking rests as necessary | □ | □ | □ | □ | □ |
|  | Going up & down a flight of stairs without using a handrail | □ | □ | □ | □ | □ |
|  | Running a short distance, such as to catch a bus | □ | □ | □ | □ | □ |
|  | Reaching overhead while standing, as if to pull a light cord | □ | □ | □ | □ | □ |
|  | Sitting down in and standing up from a low, soft couch | □ | □ | □ | □ | □ |
|  | Putting on and taking off a coat or jacket | □ | □ | □ | □ | □ |
|  | Reaching behind your back as if to put a belt through a belt loop | □ | □ | □ | □ | □ |
|  | Stepping up and down from a curb | □ | □ | □ | □ | □ |
|  | Opening a heavy, outside door | □ | □ | □ | □ | □ |
|  | Rip open a package of snack food (e.g. cellophane wrapping on crackers) using only your hands | □ | □ | □ | □ | □ |
|  | Pouring from a large pitcher | □ | □ | □ | □ | □ |
|  | Getting into and out of a car/taxi (sedan) | □ | □ | □ | □ | □ |
|  | Hiking a couple of kilometres on uneven surfaces, including hills | □ | □ | □ | □ | □ |
|  | Going up and down 3 flights of stairs using a handrail | □ | □ | □ | □ | □ |
|  | Picking up a kitchen chair and moving it, in order to clean | □ | □ | □ | □ | □ |
|  | **How much difficulty do you have…?**  **(Remember, this is without the help of someone else and without the use of any assistive walking device.)** | **5) None** | **4) A Little** | **3) Some** | **2) Quite a Lot** | **1) Cannot do** |
|  |  |  |  |  |  |  |
|  | Using a step stool to reach into a high cabinet | □ | □ | □ | □ | □ |
|  | Making a bed, including spreading and tucking in bed sheets | □ | □ | □ | □ | □ |
|  | Carrying something in both arms while climbing a flight of stairs (e.g. laundry basket) | □ | □ | □ | □ | □ |
|  | Bending over from a standing position to pick up a piece of clothing from the floor | □ | □ | □ | □ | □ |
|  | Walking around one floor of your home, taking into consideration thresholds, doors, furniture, and a variety of floor coverings | □ | □ | □ | □ | □ |
|  | Getting up from the floor (as if you were laying on the ground) | □ | □ | □ | □ | □ |
|  | Washing dishes, pots, and utensils by hand while standing at sink | □ | □ | □ | □ | □ |
|  | Walking several blocks | □ | □ | □ | □ | □ |
|  | Taking a 1.6 kilometres, brisk walk without stopping to rest | □ | □ | □ | □ | □ |
|  | Stepping on and off a bus | □ | □ | □ | □ | □ |
|  | Walking on a slippery surface outdoors | □ | □ | □ | □ | □ |

**For those who use walking devices**

|  | **When you use your cane, walker, or other walking device, how much difficulty do you have…?** | | **5) None** | | **4) A Little** | | **3) Some** | | **2) Quite a Lot** | | **1) Cannot do** | |  |
| --- | --- | --- | --- | --- | --- | --- | --- | --- | --- | --- | --- | --- | --- |
|  |  | |  | |  | |  | |  | |  | |  |
|  | | Walking 1.6 kilometres, taking rests as necessary | | □ | | □ | | □ | | □ | | □ | |
|  | | Going up and down a flight of stairs outside, without using a handrail | | □ | | □ | | □ | | □ | | □ | |
|  | | Stepping up and down from a curb | | □ | | □ | | □ | | □ | | □ | |
|  | | Opening a heavy, outside door | | □ | | □ | | □ | | □ | | □ | |
|  | | **When you use your cane, walker, or other walking device, how much difficulty do you have…?** | | **5) None** | | **4) A Little** | | **3) Some** | | **2) Quite a Lot** | | **1) Cannot do** | |
|  | | Walking around one floor of your home, taking into consideration thresholds, doors, furniture, and a variety of floor coverings | | □ | | □ | | □ | | □ | | □ | |
|  | | Walking several blocks | | □ | | □ | | □ | | □ | | □ | |
|  | | Taking a 1.6 kilometres, brisk walk without stopping to rest | | □ | | □ | | □ | | □ | | □ | |
|  | | Walking on a slippery surface outdoors | | □ | | □ | | □ | | □ | | □ | |

# Symptoms

|  | **In the past 1 month, have you been bothered a lot by any of the following problems?** | **0) No** | **1) Yes** |
| --- | --- | --- | --- |
|  | Stomach pain | □ | □ |
|  | Back pain | □ | □ |
|  | Pain in joints (arms, knees, hips, etc.) | □ | □ |
|  | Headaches | □ | □ |
|  | Chest pain | □ | □ |
|  | Feeling your heart pound or race | □ | □ |
|  | Shortness of breath/breathlessness | □ | □ |
|  | Dizziness / fainting spells | □ | □ |
|  | Nausea, bloating, indigestion or loss of appetite | □ | □ |
|  | Constipation, loose bowels, or diarrhoea | □ | □ |

# Nutrition – D) Mini Nutritional Assessment (MNA)


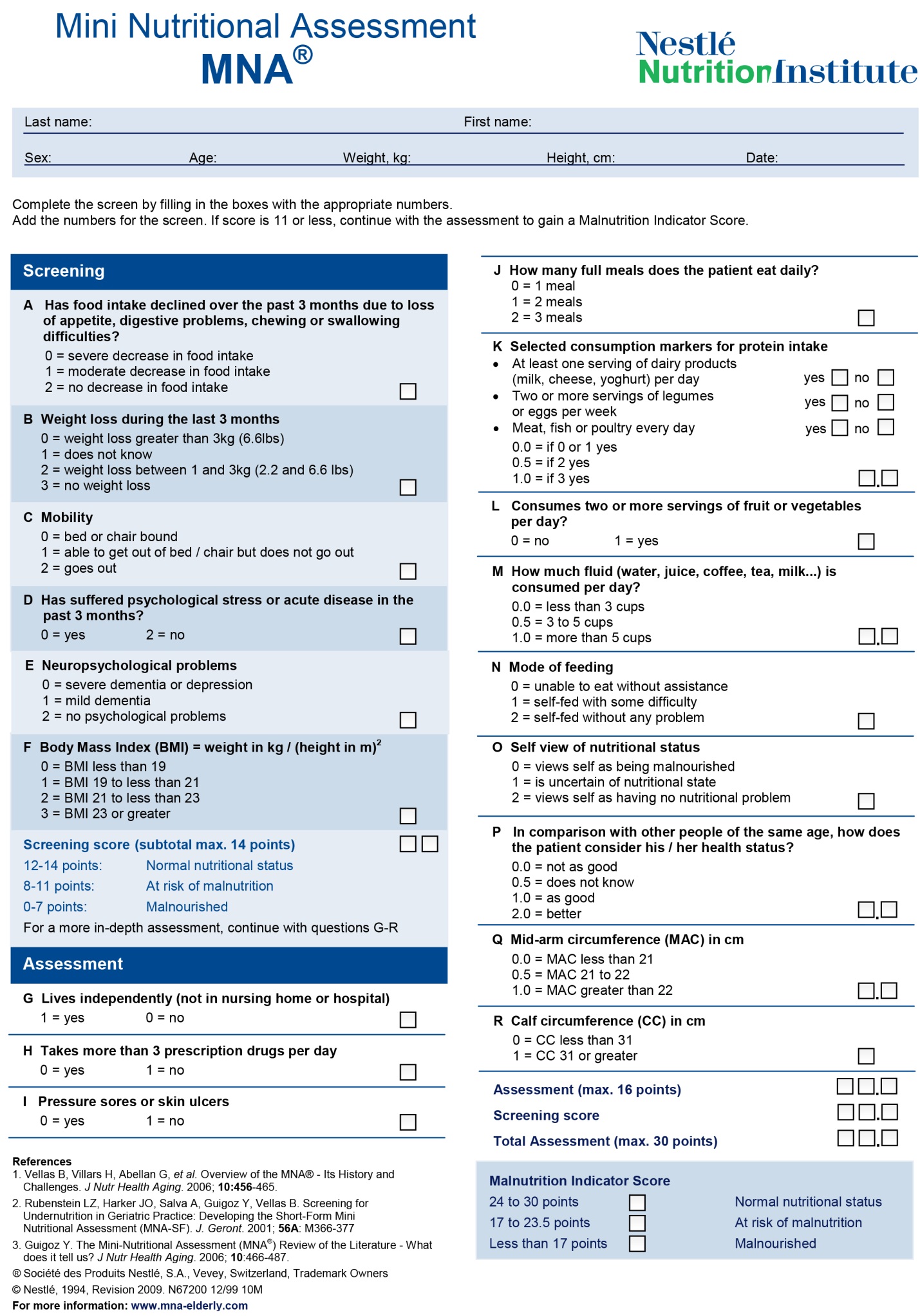


**91**

**92**

**93**

**94**

**95**

**96**

**97**

**98**

**99**

**100**

**101**

**102**

**103**

**104**

**105**

**106**

**107**

**108**

| **109.** | **In the past 6 months, how many times have you had:** |  |
| --- | --- | --- |
| **If respondent does not know his/her height, use alternative measurement below.** | | |
| **109.** | **Half-arm span (cm) / Forearm length (cm):**  [circle measurement used] |  |
|  |  |  |
| **To measure for ALL respondents:** | | |
| **110.** | **Waist circumference (cm):** |  |
| **111.** | **Hip circumference (cm):** |  |

# Healthcare Utilisation & Medication

| **112.** | **In the past 6 months, how many times have you had:** |  |
| --- | --- | --- |
|  | General Practitioner/Polyclinic visits | times |
|  | Specialist Outpatient Clinic visits | times |
|  | Emergency Department visits | times |
|  | Hospital admissions | times |
|  | TCM clinic visits |  |
|  | 1. Acupuncture | times |
|  | 1. Herbal medicine | times |

| **113.** | **Number of prescribed medications taken regularly for your chronic diseases:** |  |
| --- | --- | --- |

# Mental health

## Patient Health Questionnaire

|  | | **Over the last 2 weeks, how often have you been bothered by any of the following problems?** | **0)Not at all** | | | **1)Several days** | | | | **2)More than**  **half the days** | | | | **3)Nearly every day** | |  |  |  |
| --- | --- | --- | --- | --- | --- | --- | --- | --- | --- | --- | --- | --- | --- | --- | --- | --- | --- | --- |
|  | | 1. Little interest or pleasure in doing things | □ | | | □ | | | | □ | | | | □ | |  |  |  |
|  | | 1. Feeling down, depressed, or hopeless | □ | | | □ | | | | □ | | | | □ | |  |  |  |
|  | | 1. Trouble falling or staying asleep, or sleeping too much | □ | | | □ | | | | □ | | | | □ | |  |  |  |
|  | | 1. Feeling tired or having little energy | □ | | | □ | | | | □ | | | | □ | |  |  |  |
|  | | 1. Poor appetite or overeating | □ | | | □ | | | | □ | | | | □ | |  |  |  |
|  | | 1. Feeling bad about yourself — or that you are a failure or have let yourself or your family down | □ | | | □ | | | | □ | | | | □ | |  |  |  |
|  | | 1. Trouble concentrating on things, such as reading the newspaper or watching television | □ | | | □ | | | | □ | | | | □ | |  |  |  |
|  | | 1. Moving or speaking so slowly that other people could have noticed Or the opposite — being so fidgety or restless that you have been moving around a lot more than usual | □ | | | □ | | | | □ | | | | □ | |  |  |  |
|  | | 1. Thoughts that you would be better off dead or of hurting yourself in some way | □ | | | □ | | | | □ | | | | □ | |  |  |  |
|  | **Questions about anxiety.** | | | | **0)No** | | | **1)Yes** | | | |  | | | | | |  |
|  | 1. In the last 4 weeks, have you had an anxiety attack - suddenly feeling fear or panic? | | | | □ | | | □ | | | |  | | | | | |  |
|  | **If “NO” was checked for Q115 a), please go to question 117.** | | | | | | | | | | |  | | | | | |  |
|  | 1. Has this ever happened before? | | | | □ | | | □ | | | |  | | | | | |  |
|  | 1. Do some of these attacks come suddenly out of the blue - that is, in situations where you don’t expect to be nervous or uncomfortable? | | | | □ | | | □ | | | |  | | | | | |  |
|  | 1. Do these attacks bother you a lot or are you worried about having another attack? | | | | □ | | | □ | | | |  | | | | | |  |
|  | **Think about your last bad anxiety attack.** | | | | **0)No** | | | | **1)Yes** | | | |  | | | | | |
|  | 1. Were you short of breath? | | | | □ | | | | □ | | | |  | | | | | |
|  | 1. Did your heart race, pound, or skip? | | | | □ | | | | □ | | | |  | | | | | |
|  | 1. Did you have chest pain or pressure? | | | | □ | | | | □ | | | |  | | | | | |
|  | 1. Did you sweat? | | | | □ | | | | □ | | | |  | | | | | |
|  | 1. Did you feel as if you were choking? | | | | □ | | | | □ | | | |  | | | | | |
|  | 1. Did you have hot flashes or chills? | | | | □ | | | | □ | | | |  | | | | | |
|  | 1. Did you have nausea or an upset stomach, or the feeling that you were going to have diarrhoea? | | | | □ | | | | □ | | | |  | | | | | |
|  | 1. Did you feel dizzy, unsteady, or faint? | | | | □ | | | | □ | | | |  | | | | | |
|  | 1. Did you have tingling or numbness in parts of your body? | | | | □ | | | | □ | | | |  | | | | | |
|  | 1. Did you tremble or shake? | | | | □ | | | | □ | | | |  | | | | | |
|  | 1. Were you afraid you were dying? | | | | □ | | | | □ | | | |  | | | | | |
|  | **Over the last 4 weeks, how often have you been bothered by any of the following problems?** | | | **0)Not at all** | | | **1)Several days** | | | | **2)More than**  **half the days** | | | |  | | |  |
|  | 1. Feeling nervous, anxious, on edge, or worrying a lot about different things. | | | □ | | | □ | | | | □ | | | |  | | |  |
|  | 1. Feeling restless so that it is hard to sit still. | | | □ | | | □ | | | | □ | | | |  | | |  |
|  | 1. Getting tired very easily. | | | □ | | | □ | | | | □ | | | |  | | |  |
|  | 1. Muscle tension, aches, or soreness. | | | □ | | | □ | | | | □ | | | |  | | |  |
|  | 1. Trouble falling asleep or staying asleep. | | | □ | | | □ | | | | □ | | | |  | | |  |
|  | 1. Trouble concentrating on things, such as reading a book or watching TV. | | | □ | | | □ | | | | □ | | | |  | | |  |
|  | 1. Becoming easily annoyed or irritable. | | | □ | | | □ | | | | □ | | | |  | | |  |

# Cognition

# Montreal Cognitive Assessment (MOCA)


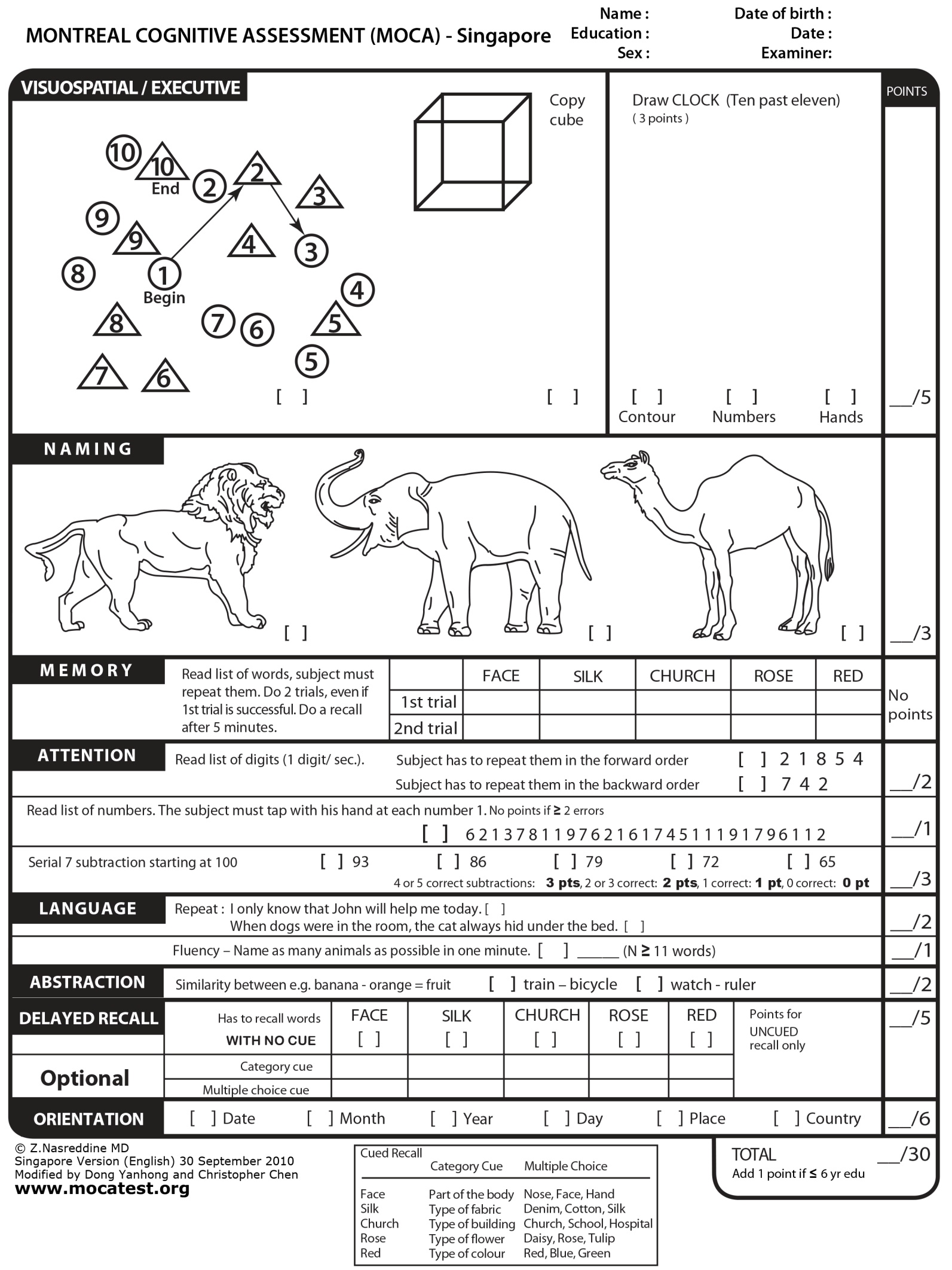


**118. VISUOSPATIAL / EXECUTIVE**

**119. NAMING**

**120. MEMORY**

**121. ATTENTION**

**122. LANGUAGE**

**123. ABSTRACTION**

**124. DELAYED**

**RECALL**

**125. ORIENTATION**

# Socio-economic status

|  |  | **0) No** | **1) Yes** |  |
| --- | --- | --- | --- | --- |
| **126.** | **Do you often run out of money, even with proper spending plan, to buy essential items or pay bills to maintain basic living needs?** (i.e. Accommodation, food, transportation and healthcare)? | □ | □ |  |

Social

## Social isolation

| **127.** | **How many relatives/friends do you see/hear from at least once a month?** | **0) 0** | **1) 1** | **2) 2** | **3) 3-4** | **4) 5-8** | **5) ≥ 9** |  |  |
| --- | --- | --- | --- | --- | --- | --- | --- | --- | --- |
|  | Relatives (including children, grandchildren, in-laws, siblings, cousins, etc.) | □ | □ | □ | □ | □ | □ |  |  |
|  | Friends | □ | □ | □ | □ | □ | □ |  |  |
| **128.** | **How many relatives/friends do you feel at ease with whom you can talk about private matters?** | **0) 0** | **1) 1** | **2) 2** | **3) 3-4** | **4) 5-8** | **5) ≥ 9** |  |  |
|  | Relatives (including children, grandchildren, in-laws, siblings, cousins, etc.) | □ | □ | □ | □ | □ | □ |  |  |
|  | Friends | □ | □ | □ | □ | □ | □ |  |  |
| **129.** | **How many relatives/friends do you feel close to such that you could call on them for help?** | **0) 0** | **1) 1** | **2) 2** | **3) 3-4** | **4) 5-8** | **5) ≥ 9** |  |  |
|  | Relatives (including children, grandchildren, in-laws, siblings, cousins, etc.) | □ | □ | □ | □ | □ | □ |  |  |
|  | Friends | □ | □ | □ | □ | □ | □ |  |  |

## Loneliness

|  |  | 1. **Hardly ever** | **2) Some of**  **the time** | **3) Often** |  |
| --- | --- | --- | --- | --- | --- |
| **130.** | **How often do you feel that you lack companionship?** | □ | □ | □ |  |
| **131.** | **How often do you feel left out?** | □ | □ | □ |  |
| **132.** | **How often do you feel isolated from others?** | □ | □ | □ |  |

## Quality of life – I) EQ-5D-5L

**Under each heading, please tick the ONE box that best describes your health TODAY**

|  | **Mobility** | | | | |  |  | |
| --- | --- | --- | --- | --- | --- | --- | --- | --- |
|  | 1) I have no problems in walking about | | | | | □ |  | |
|  | 2) I have slight problems in walking about | | | | | □ |  | |
|  | 3) I have moderate problems in walking about | | | | | □ |  | |
|  | 4) I have severe problems in walking about | | | | | □ |  | |
|  | 5) I am unable to walk about | | | | | □ |  | |
|  | **Self-care** | | | | |  |  | |
|  | 1) I have no problems washing or dressing myself | | | | | □ |  | |
|  | 2) I have slight problems washing or dressing myself | | | | | □ |  | |
|  | 3) I have moderate problems washing or dressing myself | | | | | □ |  | |
|  | 4) I have severe problems washing or dressing myself | | | | | □ |  | |
|  | 5) I am unable to wash or dress myself | | | | | □ |  | |
|  | **Usual activities (e.g. Work, study, housework, family or leisure activities)** | | | | | | | |
|  | 1) I have no problems doing my usual activities | | | | | □ |  | |
|  | 2) I have slight problems doing my usual activities | | | | | □ |  | |
|  | 3) I have moderate problems doing my usual activities | | | | | □ |  | |
|  | 4) I have severe problems doing my usual activities | | | | | □ |  | |
|  | 5) I am unable to do my usual activities | | | | | □ |  | |
|  | **Pain / discomfort** | | | | |  |  | |
|  | 1) I have no pain or discomfort | | | | | □ |  | |
|  | 2) I have slight pain or discomfort | | | | | □ |  | |
|  | 3) I have moderate pain or discomfort | | | | | □ |  | |
|  | 4) I have severe pain or discomfort | | | | | □ |  | |
|  | 5) I have extreme pain or discomfort | | | | □ | |  | |
|  | **Anxiety / depression** | | |  | | | |  |
|  | 1) I am not anxious or depressed | | | □ | | | |  |
|  | 2) I am slightly anxious or depressed | | | □ | | | |  |
|  | 3) I am moderately anxious or depressed | | | □ | | | |  |
|  | 4) I am severely anxious or depressed | | | □ | | | |  |
|  | 5) I am extremely anxious or depressed | | | □ | | | |  |
|  | **We would like to know how good or bad your health is TODAY.**   - This scale is numbered from 0 to 100. - 100 means the best health you can imagine. 0 means the worst health you can imagine. - Mark an X on the scale to indicate how your health is TODAY. - Now, please write the number you marked on the scale in the box below: | | | | |  | | |
|  | YOUR HEALTH TODAY = |  |  | | |  |  |  |
|  |  |  |  | | |  |  |  |

~~~~~~~~~~ END OF SURVEY ~~~~~~~~~~
